# The Physical Activity and Exercise as Key Role Topic in Sports Medicine for Old People Quality of Life

**DOI:** 10.3390/medicina58060797

**Published:** 2022-06-13

**Authors:** Pedro Forte, António M. Monteiro

**Affiliations:** 1 Department of Sports, Higher Institute of Educational Sciences of the Douro (ISCE Douro), 4560-708 Penafiel, Portugal; 2 Department of Sport Sciences and Physical Education, Instituto Politécnico de Bragança (IPB), 5300-253 Braganza, Portugal; mmonteiro@ipb.pt; 3 Research Center in Sports Sciences, Health and Human Development (CIDESD), 6201-001 Covilha, Portugal; 4 CI-ISCE, ISCE Douro, 4560-708 Penafiel, Portugal

## 1. Ageing, Body Composition and Physical Activity

The body composition varies with ageing. There are phases where the body grows, fills (increase in body mass), maintaining, and, finally, declining of the body mass (essentially derived from aging). The elderly population has a important representation in age pyramids. Furthermore, this population has come to have a greater representation in the age pyramids (essentially developed countries). The age-related changes over time lead to reduced adaptability, changes in functional capacity (such as autonomy and independency) and even, eventually, death [1]. 

The sedentarism is associated with the decline of physiological systems. The diseases increase, the decreasing autonomy, independency and quality of life are typically observed in aged populations [2]. These mostly result in higher disability levels, dependency, and higher probability of diseases incidence [3]. With low levels of physical activity, the increase in body fat levels is the main body composition outcome. Normally, this increase in body fat is also characterized by an increase in abdominal circumference and reduction in lean mass (essentially a decrease in muscle mass) [4].

The association between physical activity, exercise and positive health benefits has been clear [5], with a consensus that individuals physically active seem to enjoy a longer and better quality life in comparison to less active individuals [1,6]. Additionally, the physically active lifestyles have been associated with better functioning of the cardiovascular, respiratory, and muscular systems, as well as reduced risks of morbidity and mortality. These health-related problems have been identified in obese people with chronic diseases. 

Regarding physiology, we found scientific evidence in favour of the contribution of physical activity as therapeutic strategy to prevent the functional decrease in the cardiovascular system, sarcopenia, loss in the bone mineral mass density, increased blood pressure, reduced insulin resistance and glucose intolerance, and increased serum triglyceride levels. Additionally, the physical activity and exercise positively influence age-related changes in metabolism function; has a protective role against unfavourable changes in body composition, reducing weight gain, and fat mass and increasing fat-free mass [6,7].

## 2. Ageing, Physical Fitness and Independency

Ageing is associated with a decline in physiological systems and physical capacity [8] and increase in the prevalence of diseases [9]. Additionally, a decrease in the autonomy and quality of life of the elderly population is typically observed [10]. It is important to highlight the difficulties of the elderly people to perform the daily life activities, where the physical fitness, function, and skills are necessary for the independency. The functional fitness, the muscle strength, joint flexibility, body balance, and cardiovascular capacity, plays an important role in preserving elderlies’ autonomy [8,11,12].

With aging, it is possible to note neuromuscular changes. Typically, the decline in muscle mass, strength, endurance, and power, as well as in motor coordination. Mostly, the reaction and movement speed capacity are reduced [13]. These changes, result in slower movements, functional limitations in gait, weight transfer activities, and static and dynamic balance [8]. In addition to functional losses, these neuromuscular alterations increase the risk of falls and consequent fractures by the bone reduced mineral density. Additionally, due to the physical and functional losses, the loss of mobility and the falls trend to increase. That may result in psychological and social consequences, such as anxiety, depression, and fear. Moreover, it may lead to physical activity self-restriction and the consequent loss of autonomy [6,8]. 

The literature have shown that the physical-functional decline due ageing can be partially reversed, or at least minimized by physical exercise [14]. However, most studies rely on specific intensive training protocols for isolated functions and in most cases, use aerobic resistance training and/or strength training [11]. Lately, the main recommendations advise combining aerobic training with strength, flexibility, and balance training [8,11,12].

The health-related physical fitness involves fitness components that affect habitual physical activity and are related to health status, such as cardiorespiratory fitness, muscular fitness, flexibility, motor coordination, and body composition [12]. Regarding the old people physical fitness, their functionality is a very important topic regarding health-related issues, autonomy and independence. Rikli and Jones [14] argue that for the elderlies to be able to autonomously perform everyday tasks, such as shopping, transporting objects, and dressing, it is crucial that they maintain a minimum level of physical fitness. 

The exercise is believed to be the best fall prevention strategy in the elderlies. Additionally, it prevents the mobility, balance, and muscle strength reduction [8]. Additionally, contributes to physical and physiological improvement, such as positive psychological effects, particularly the self-esteem and well-being [15]. Furthermore, through physical exercise it is possible to reduce the fear of falling and consequently ensure greater autonomy and a feeling of well-being in the elderly. 

## 3. Research Topics

Improving the physical fitness by physical activity or exercise programs may change the body composition and independency. Additionally, the higher the independency levels, the better the quality of life, based on psychophysiological approach. Based upon this, there is a need for research aiming to simplify exercise training programs and provide guidelines for prescription to improve physical fitness, body composition, and quality of life. Reducing the fear and risk of falls, minimizing morbidity controlling specific diseases with exercise therapy will contribute to improve the health-related quality of life of old people. Research articles regarding different strategies and programs are required to improve the scientific knowledge and provide more evidence-based approaches about the effects of physical activity and exercise programs for old people quality of life. However, exercise and physical activity should be the key role for old people sports medicine research.

## Data Availability

Not applicable.

## References

[1] Spirduso W.W., Francis K.L., MacRae P.G. (1995). Physical Dimensions of Aging.

[2] Lian T.C., Bonn G., Han Y.S., Choo Y.C., Piau W.C. (2016). Physical Activity and Its Correlates among Adults in Malaysia: A Cross-Sectional Descriptive Study. PLoS ONE.

[3] Walter S., MacKenbach J., Vokó Z., Lhachimi S., Ikram M.A., Uitterlinden A.G., Newman A.B., Murabito J.M., Garcia M.E., Gudnason V. (2012). Genetic, Physiological, and Lifestyle Predictors of Mortality in the General Population. Am. J. Public Health.

[4] Asp M., Simonsson B., Larm P., Molarius A. (2017). Physical Mobility, Physical Activity, and Obesity among Elderly: Findings from a Large Population-Based Swedish Survey. Public Health.

[5] Vogel T., Brechat P.H., Leprêtre P.M., Kaltenbach G., Berthel M., Lonsdorfer J. (2009). Health Benefits of Physical Activity in Older Patients: A Review. Int. J. Clin. Pract..

[6] Monteiro A.M., Silva P., Forte P., Carvalho J. (2019). The Effects of Daily Physical Activity on Functional Fitness, Isokinetic Strength and Body Composition in Elderly Community-Dwelling Women. J. Hum. Sports Exerc..

[7] Fletcher G., Trejo J.F. (2005). Why and How to Prescribe Exercise: Overcoming the Barriers. Clevel. Clin. J. Med..

[8] Monteiro A.M., Forte P., Carvalho J., Barbosa T.M., Morais J.E. (2021). Relationship between Fear of Falling and Balance Factors in Healthy Elderly Women: A Confirmatory Analysis. J. Women Aging.

[9] Maggi S., Noale M., Gallina P., Bianchi D., Marzari C., Limongi F., Crepaldi G. (2006). Metabolic Syndrome, Diabetes, and Cardiovascular Disease in an Elderly Caucasian Cohort: The Italian Longitudinal Study on Aging. J. Gerontology. Ser. A Biol. Sci. Med. Sci..

[10] Bean J.F., Vora A., Frontera W.R. (2004). Benefits of Exercise for Community-Dwelling Older Adults. Arch. Phys. Med. Rehabil..

[11] Monteiro A.M., Bartolomeu R.F., Forte P., Carvalho J. (2019). The Effects of Three Different Types of Training in Functional Fitness and Body Composition in Older Women. J. Sport Health Res..

[12] Monteiro A.M., Forte P., Carvalho M.J. (2020). The Effect of Three Different Training Programs in Elderly Women’s Isokinetic Strength. Motricidade.

[13] Doherty T.J. (2003). Invited Review: Aging and Sarcopenia. J. Appl. Physiol..

[14] Rikli R., Jone J. (2013). Senior Fitness Test Manual.

[15] Daley M.J., Spinks W.L. (2012). Exercise, Mobility and Aging. Sports Med..

